# PARP inhibitor olaparib sensitizes esophageal carcinoma cells to fractionated proton irradiation

**DOI:** 10.1093/jrr/rrz088

**Published:** 2020-01-24

**Authors:** Shun-ichiro Kageyama, Du Junyan, Hidehiro Hojo, Atsushi Motegi, Masaki Nakamura, Katsuya Tsuchihara, Tetsuo Akimoto

**Affiliations:** National Cancer Center Hospital East, 6-5-1 Kashiwanoha, Kashiwa, Chiba 277-8577, Japan

**Keywords:** esophageal cancer, proton, radiation, PARP, BRCA

## Abstract

Proton beam therapy (PBT) combined with chemotherapy, such as *cis*-diamminedichloroplatinum (II) (CDDP) and 5-fluorouracil (5-FU), has been employed as an alternative approach to improve clinical outcomes. PBT has been reported to be effective against esophageal cancer. However, apart from 5-FU and CDDP, almost no other drug has been tested in combined chemotherapy with PBT. Therefore, we investigated the effects of a poly (ADP-ribose) polymerase inhibitor on enhancing proton beam effects using esophageal cancer cell lines that exhibit resistance to radiation and CDDP. Esophageal squamous cell carcinoma cell lines OE-21 and KYSE-450 were exposed to the drugs for 1 h prior to irradiation. The cell survival curve was obtained using a clonogenic assay and the sensitizing effect ratio (SER) was calculated. The clonogenic assay was used to compare the effect of multi-fractioned irradiation between 8 Gy/1 fraction (fr) and 8 Gy/4 fr. γH2AX, Rad51, BRCA1, BRCA2 and 53BP1 foci were detected via immunofluorescence. Olaparib exhibited an SER of 1.5–1.7 on PBT. The same sensitizing effect was exhibited in multi-fractioned irradiation, and the combined use increased the expression of double-strand breaks and homologous recombination-related genes in an additive manner. Such additive effects were not observed on non-homologous end joining-related genes. We demonstrated that olaparib has a high sensitizing effect on PBT in platinum- and radiation-resistant esophageal cancer cells. Our results suggest a potential clinical application of olaparib-proton irradiation (PT) against platinum- and radiation-resistant esophageal cancer.

## INTRODUCTION

### Chemoradiotherapy for esophageal cancer

The efficacy of chemoradiotherapy in combination with 5-fluorouracil (5-FU) and *cis-*diamminedichloroplatinum (II) (CDDP) was demonstrated in 1999; since then, it has served as a standard treatment for esophageal cancer [1, 2]. Thus far, phase III trials that significantly extend survival compared with combined 5-FU, CDDP and radiotherapy (FP-RT) have not been reported.

Several combination therapies involving taxan, a cytotoxic drug that targets microtubules, and gefitinib and cetuximab, two drugs that target the EGFR, have been tested. However, these drugs have not displayed superiority to FP-RT in clinical trials [3, 4]. Therefore, finding an alternative approach for treating esophageal cancer refractory to FP-RT continues to be a challenge [2, 5].

Because the esophagus is a centrally located thoracic structure, there must be a balance between delivering the cytotoxic agent to the target at an appropriately high dose and minimizing the dose to nearby critical structures. Excessive radiation received by these critical structures, particularly the heart and lungs, may lead to clinically significant toxicities, including pneumonitis, pericarditis and myocardial infarction. Although technological advancements in photon RT delivery, such as intensity-modulated RT, have decreased the risk of such toxicities, mounting evidence indicates that further risk reductions can be achieved with proton beam therapy (PBT) [6]. However, reports on photon therapy are much more common than reports on drugs that exhibit radiosensitizing effects. Currently, chemotherapy combined with PBT uses therapies that have previously been used in combination with photon therapy, such as CDDP and 5-FU, and are not based on clear evidence. Therefore, the elucidation of sensitizers and their mechanisms in the context of proton beams is necessary. 

**Fig. 1:**
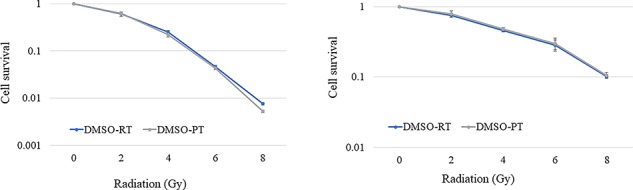
OE-21 and KYSE-450 cell survival after photon and proton irradiation. Clonogenic cell survival assays were performed in triplicate after irradiation with 2, 4, 6 or 8 Gy per fraction. Values correspond to the mean ± standard deviation. Data analysis was performed on pooled values from at least three independent experiments.

**Fig. 2:**
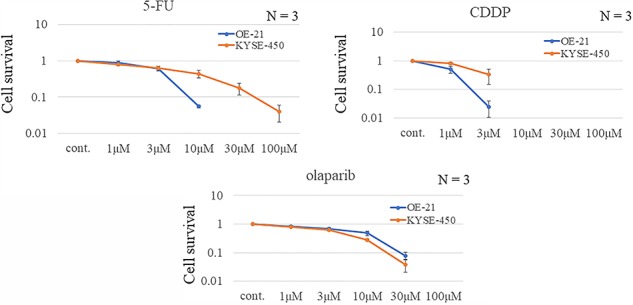
OE-21 and KYSE-450 cell survival after treatment with DNA-damaging agents. Clonogenic cell survival assays were performed in duplicate. Values correspond to the mean ± standard deviation. Data analysis was performed on pooled values from at least three independent experiments.

**Fig. 3:**
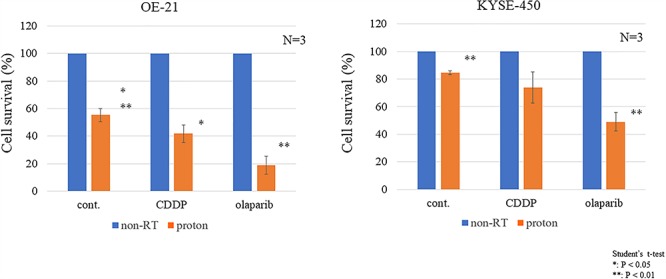
The colony-forming ability in OE-21 and KYSE-450 cells upon radiation was compared with that of the control group and the drug-only group. ^*^P < 0.05 (Student’s *t*-test). ^**^P < 0.01 (Student’s *t*-test). Cont., control. Non-RT., not irradiated and treated by drug only.

DNA-damaging agents have recently been reported to possess a novel mechanism of action [7, 8]. The poly (ADP-ribose) polymerase (PARP) family of proteins can convert single-strand breaks (SSBs) into double-strand breaks (DSBs), which are amenable to repair by homologous recombination (HR). Accordingly, PARP inhibitors can induce synthetic lethality in cancer cells having weak HR repair abilities, such as BRCA-mutated cancers. Recently, PARP inhibitors have been shown to exhibit high radiosensitizing effects in prostate cancer, pancreatic cancer and breast cancer cell lines [6, 8]. An increasing number of studies have investigated these differences which cause different biological effect between proton and photon in detail at the cellular and molecular levels [9]. Photon-triggered DSBs are primarily repaired by non-homologous end joining (NHEJ), whereas proton-induced DSBs are repaired by HR [10]. Protons and PARP inhibitors, which both stimulate HR-dependent DSB repair, are therefore of particular therapeutic relevance because they may exhibit a strong sensitizing effect.

Olaparib is an FDA-approved drug that was recently reported to exhibit sensitization in pancreatic cancer and lung adenocarcinoma cell lines [11]. In Japan, olaparib and PBT received insurance approval in 2018, and expansion of its adaptation is expected in the future. Comprehensive analyses suggest that esophageal cancer displays abnormalities in DSB repair pathways such as PARP and BRCA. In a TCGA dataset, we found that 8.2% have BRCA1 and BRCA2 mutations or copy number alterations and 1.5% of patients have PARP1 copy number alterations (see online supplementary Figure S1). Furthermore, the other genes, such as ATR and Rad 51, that are necessary to repair DNA damage by irradiation also have mutations or copy number alterations. Therefore, treatments that target DSBs are expected. However, studies that compare fractionated irradiation with standard therapies such as 5-FU and CDDP and molecular mechanisms are rare; this information would provide the rationale for clinical trials.

In the present study, we demonstrated the effect of PBT combined with olaparib on esophageal cancer cell lines and investigated the underlying mechanism of this method to establish an effective treatment for platinum- and radiation-resistant esophageal cancer, which is of clinical relevance.

## MATERIALS AND METHODS

### Cell lines and culture

Human esophageal cancer cell lines OE21 and KYSE450 were obtained from the cell banks of Public Health England (Salisbury, UK) and the National Institutes of Biomedical Innovation (Osaka, Japan), respectively; they were used within 20 passages for the present experiments [12]. The cells were maintained in RPMI 1640 medium (SIGMA-ALDRICH, Saint Louis, MO, USA) containing filtered 10% fetal bovine serum (Biowest, Nuaillé, France). Both cell lines were incubated under 100% humidity in the presence of 5% CO_2_ at 37°C. An authentication of the cell lines OE-21 and KYSE 450 was confirmed by STR profiling (data not shown).

### Photon and proton irradiation

To perform photon irradiation, both cell lines were irradiated with a 6-MV X-ray beam at a dose rate of 6 Gy/min using a Linac (Varian Medical Systems, Palo Alto, CA, USA) at the National Cancer Center Hospital East (NCCNE) as previously described [13]. To perform proton irradiation (PT), both cell lines were irradiated with a 235-MeV proton beam (Sumitomo Heavy Industry, Co, Ltd., Kanagawa, Japan), also at the NCCNE. Proton beams dispersed by a double-scattering method were shaped down with a brass collimator to irradiate a field size of 20 × 20 cm. The depth of irradiation was precisely modulated by the placement of polyethylene plates of appropriate thickness, based on a water equivalent thickness of 3 mm, which was calculated from the incident side’s thickness of the flask or dish and the medium at a position between the collimator and the sample. The field size was 15 × 20 cm and the flask surface dose homogeneity was ≥95%. The cells were seeded in 25-cm^2^ flasks (Corning, NY, USA) or 96-well dishes (Corning, New York, USA) and placed on polyethylene plates when the perpendicular proton or photon beam irradiation was performed. All experiments were conducted in triplicate. Irradiation was performed only on weekdays within a week, and once a week rest was permitted as in the clinical rule.

**Fig. 4:**
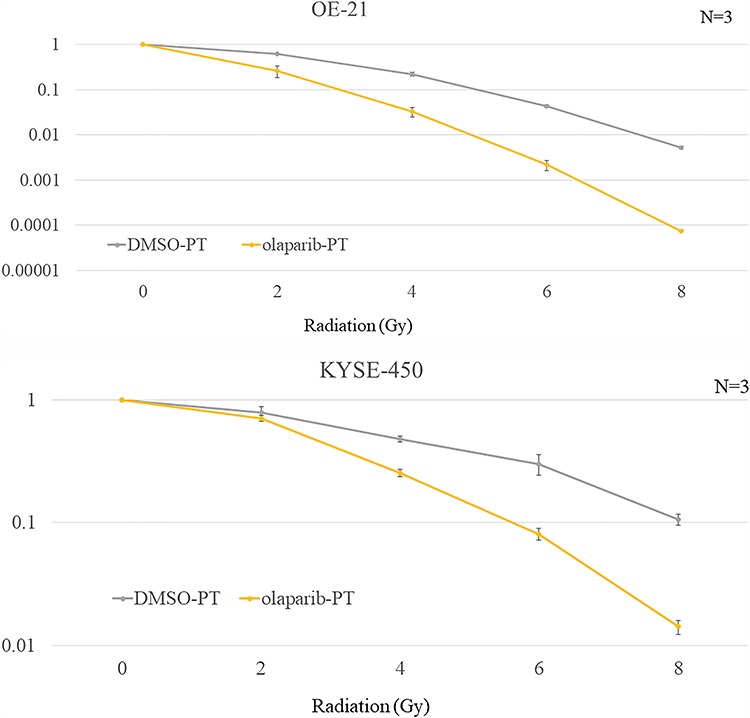
Sensitivity of OE-21 and KYSE-450 cells to proton irradiation with or without olaparib (3 μM). (**A**) OE-21 and (**B**) KYSE-450 cell survival prior to or after treatment with olaparib for 1 h before irradiation (2, 4, 6 or 8 Gy per fraction). The cells were fixed and stained with crystal violet and the number of colonies was counted in duplicate. Error bars correspond to the standard deviation of the mean. Data analysis was performed on pooled values from at least three independent experiments. DMSO = dimethyl sulfoxide, PT = proton irradiation.

**Fig. 5:**
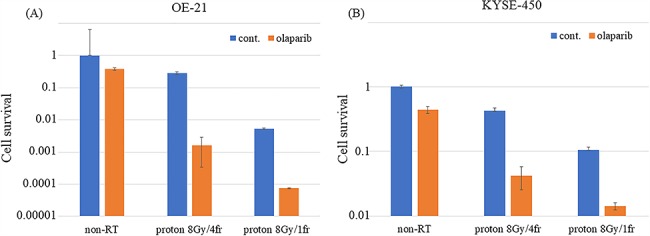
Sensitivity of OE-21 and KYSE-450 cells to multifraction proton irradiation with or without olaparib (3 μM). (**A**) OE-21 and (**B**) KYSE-450 cell survival after single or multifraction irradiation (8 Gy per four fractions) with or without prior treatment with olaparib for 1 h. The cells were fixed and stained with crystal violet and the number of colonies was counted in duplicate. Error bars correspond to the standard deviation of the mean. Data analysis was performed on pooled values from at least three independent experiments. Cont= control, Non-RT. = not irradiated and treated by drug only.

### Clonogenic assay

OE21 and KYSE450 cells were seeded in duplicate or triplicate in 25-cm^2^ tissue culture flasks containing 5 mL of the culture medium at a concentration of 400–12 800 cells per flask, depending on the irradiation dose. The flasks were incubated for 24 h prior to irradiation with the proton or photon beams and returned to the CO_2_ incubator at 37°C following irradiation. After 8 or 12 days, the colonies were fixed with 4% formalin and stained with 1% crystal violet. Colonies that contained >50 cells were counted, and the surviving fractions were calculated as the ratio of the plating efficiency of irradiated cells to that of unirradiated cells.

### Immunofluorescence analysis

The cells were seeded onto 96-well dishes at a density of 1.0 × 10^3^–3.0 × 10^3^ cells per dish for 24 h prior to irradiation and were cultured at 37°C in the presence of 5% CO_2_; cells in the plateau phase were used for all experiments in this study. Following proton or photon irradiation, the cells were incubated for 2 or 24 h, washed thrice with phosphate-buffered saline (PBS) and fixed with 4% formaldehyde for 15 min at each time point. The cells were then washed thrice with PBS for 3 min and blocked with blocking buffer containing 1% filtered bovine serum albumin (Roche, Basel, Switzerland) and 1% Triton X-100 (SIGMA-ALDRICH, Saint Louis, MO, USA) in PBS and incubated for 1 h at room temperature. The cells were then incubated with a ×100 diluted anti-rabbit antibody directed against phospho-histone H2A.X Serine139 (Cell Signaling Technology, Danvers, MA, USA), overnight at 4°C. After incubation with the primary antibody, the cells were again washed three times with PBS. Next, blocking buffer containing goat anti-rabbit Alexa Fluor 488 IgG secondary antibodies (Invitrogen, Waltham, MA, USA) was added to the cells, and the cell suspension was allowed to stand for 2 h at room temperature. The cells were again washed thrice with PBS before the addition of mounting medium containing hoechst (Vector Laboratories, Burlingame, CA, USA). Images were obtained using the Thermo Scientific ArrayScan system (Thermo Fisher, Yokohama, Japan) [14]. We use the same conditions with other protein detection. First, antibodies for Rad 51 (sc-8349), BRCA1 (sc-642), BRCA2 (sc-28235) and 53BP1 (sc-22760) (Santa Cruz Biotechnology, CA, USA) were used and the foci in each cell were counted using HCS Studio 2.0 Cell Analysis Client Software, then the mean number of foci per nucleus was calculated. More than 100–300 cells per well were evaluated following irradiation. The procedures were normalized with previously reported protocols. Immunostaining was evaluated for each antibody using a fluorescence microscope. Gamma H2AX was used to assess normality, and it was confirmed that it was radiation dose-dependent (see online supplementary Figure S2).

### Poly (ADP-ribose) polymerase inhibitor treatment

A PARP inhibitor, olaparib (AZD2281), was purchased from Selleck Chemicals (Houston, TX, USA). After the initial treatment with olaparib, olaparib was added again each time the medium was changed. In the radiation combination experiment, the drug was added 1 h before irradiation. Clonogenic survival curves and the sensitization enhancement ratio at 10% (SER_0_) were generated using OriginPro version 8.5.1 software (OriginLab Corp).

### Statistical analysis

The individual experiments were performed at least in triplicate. The statistical significance of the observed differences was analysed using Student’s *t*-test.

## RESULTS

Sensitivity of esophageal cancer cell lines to anticancer agents and radiation We confirmed that esophageal cancer cell lines OE-21 and KYSE-450 displayed resistance to photon and proton irradiation and 5-FU and CDDP, respectively (Figs. 1 and 2). Radiosensitivity was confirmed via colony assay (Fig. 1). DNA damage and drug sensitivity were assessed for the following drugs: 5-FU, an antimetabolite; CDDP, a platinum preparation; and olaparib, a PARP inhibitor. Compared with OE-21 cells, KYSE-450 cells were more resistant to 5-FU and CDDP but more sensitive to olaparib (Fig. 2).

### Radiosensitization effect of olaparib on esophageal cancer cell lines

CDDP is the most commonly used drug for enhancing radiosensitivity in esophageal cancer. The radiosensitizing effect of olaparib was compared with that of CDDP in OE-21 and KYSE-450 cells after proton irradiation with a 2-Gy dose. Next, we compared the colony-forming abilities between the control group and the drug-only group after radiation. We found that KYSE-450 cells exhibited a higher resistance to cytotoxic drug and radiation alone which are used as clinical treatment for esophageal cancer (Fig. 2). In OE-21 cells, both CDDP and olaparib significantly enhanced the radiation-induced anti-tumor effect, whereas in KYSE-450 cells, which displayed more resistance to radiation, this effect was observed only with olaparib(Fig. 3).

Next, we investigated the proton-sensitizing effect of olaparib in detail using a clonogenic assay. Olaparib exhibited a SER of 1.7 for OE-21 cells and 1.5 for KYSE-450 cells that had high radiation and CDDP resistance (Fig. 4). The effect of using multifraction 8 Gy/4 fr doses is shown in Fig. 5.

### Biochemical properties of olaparib-RT and olaparib-PT

To investigate olaparib and proton-induced DNA damage and repair of esophageal cancer cells, DNA damage after the treatment and expression of a DNA repair enzyme were examined. We used the number of γ-H2AX foci as an indicator of DNA damage. 53BP1 was used as a NHEJ reporter, and BRCA1, BRCA2 and Rad51 were used as HR markers [10, 15]. Both PBT and olaparib significantly increased the number of foci in both cell lines compared with the untreated group. In the proton-treated group, olaparib combined with PBT tended to increase the number of foci, but there was no significant difference in the OE-21 cells; conversely, the number of foci was increased significantly in the KYSE-450 cells.

**Fig. 6:**
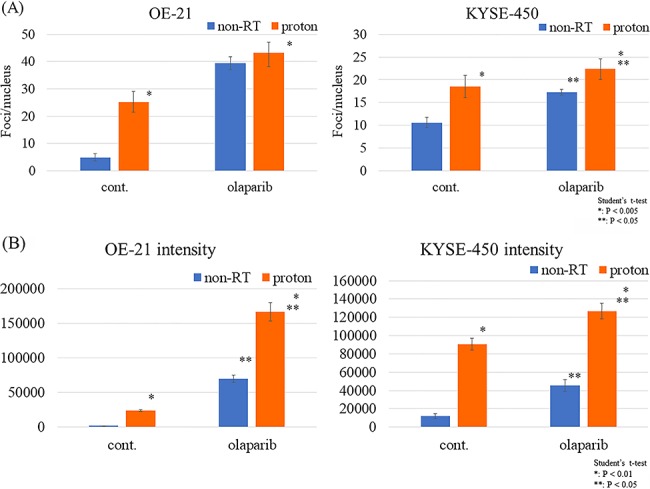
DNA damage and DNA repair gene expression after multifraction proton irradiation (8 Gy per four fractions) with olaparib. (**A**) Number of γ-H2AX foci in OE-21 and KYSE-450 cells treated with proton irradiation with or without olaparib. (**B**) Intensity and average γ-H2AX foci area in OE-21 and KYSE-450 cells treated with proton irradiation with or without olaparib. The number of foci per nucleus was counted and compared under the same conditions. Data were obtained from at least 100 cells for each condition. *P-*values (Student’s *t*-test) are indicated on the figures. Cont. = control, Non-RT. = not irradiated and treated by drug only.

In addition, we evaluated the intensity of γ-H2AX foci, which is an indicator used for DNA damage assessment and to count the number of foci (Fig. 6B) [16, 17]. The intensity showed a similar tendency to the number of foci, and the intensity evaluation indicated that a significantly higher accumulation of DNA damage was found for the combination of PBT and olaparib compared with PBT alone.

**Fig. 7:**
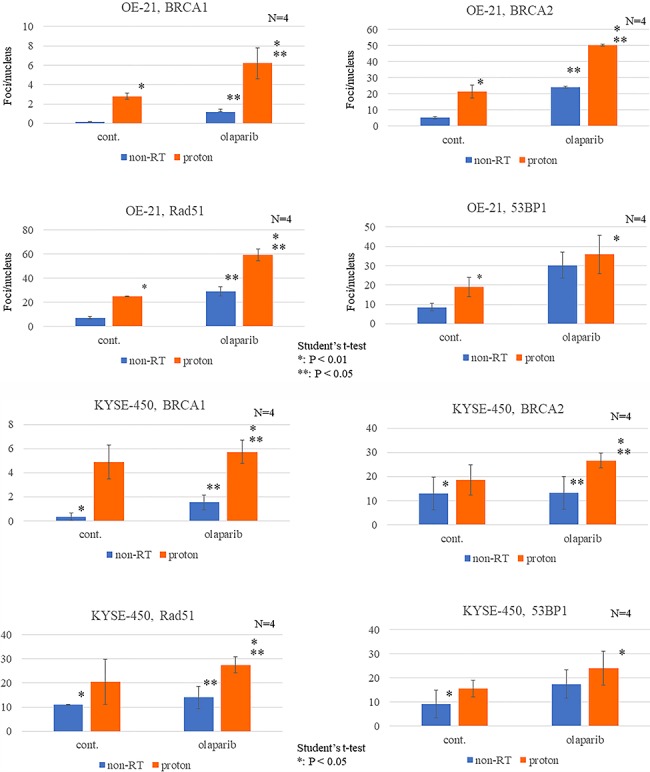
Dynamics of DNA repair enzymes after irradiation with or without olaparib. Number of BRCA1, BRCA2, Rad51 and 53BP1 foci in proton-irradiated (**A**) OE-21 and (**B**) KYSE-450 cells treated with or without olaparib (3 μM) 24 h after multifraction proton irradiation (8 Gy per four fractions). Data were obtained from at least 500 cells for each condition. *P-*values (Student’s *t*-test) are indicated on the figures.

The levels of BRCA1, BRCA2 and Rad51 were significantly higher in the olaparib-PT group than in the control and olaparib-only groups. In contrast, proton irradiation did not increase the 53BP1 expression level (Fig. 7).

**Fig. 8:**
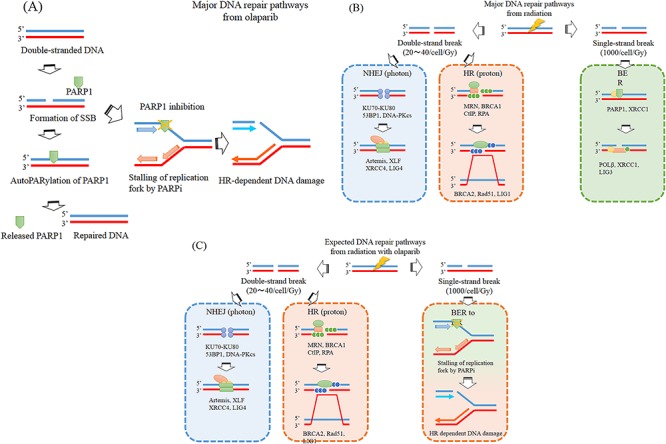
Outline of DNA damage repair pathway. Major DNA repair pathways from olaparib (**A**) and photon and proton irradiation (**B**). Expected DNA repair pathways from radiation with olaparib (**C**).

## DISCUSSION

### Comparative effect of olaparib, radiation, 5-FU and CDDP on esophageal cancer cells

In the present study, we observed that esophageal cancer cells resistant to radiation and DNA-damaging agents were sensitive to the PARP inhibitor olaparib. We examined the sensitivity to radiation alone and to the drug alone in two esophageal cancer cell lines: OE-21 and KYSE-450. In accordance with previous reports, OE-21 cells were moderately affected whereas KYSE-450 cells were highly affected by both treatments. KYSE-450 cells also displayed resistance to 5-FU and CDDP, two commonly used radiosensitizers. NHEJ is the primary pathway for repairing DNA damage caused by agents such as 5-FU and CDDP, suggesting that these cells are resistant to general DNA damage [17].

The cells were also resistant to radiation. Remarkably, the similar sensitivity displayed by OE-21 and KYSE-450 cells to olaparib was not consistent with their RT-resistance.

### Validation of olaparib as a proton radiosensitizer

We determined that olaparib has an excellent proton radiosensitizing effect in radiation and CDDP-resistant esophageal cancer cells.

The radiosensitizing effect of olaparib was compared with that of CDDP in OE-21 and KYSE-450 cells (Fig. 3). CDDP and olaparib displayed a significant effect in OE-21 cells, whereas only olaparib was effective in KYSE-450 cells. Interestingly, the SER of olaparib on PBT was 1.5–1.7, which was higher than that of a previously reported proton sensitizer [18, 19] (Fig. 4).

Our results are consistent with previous reports of RT-resistant cell lines being immune to DNA-damaging agents such as CDDP. In contrast, olaparib caused increased proton radiosensitization even in RT-resistant cell lines. Although both HR and NHEJ are important for DNA repair in response to CDDP alone, radiation alone or radiation combined with CDDP, NHEJ is believed to be the primary pathway [20, 21]. Conversely, although no repair pathway for olaparib-PT has yet been reported, it is believed that HR activity is more important for DNA damage caused by olaparib [22].

Interestingly, it has recently been reported that olaparib may be more effective in cancer cells with high NHEJ activity. The formation of a complex containing 53BP1 and Rev7 on DSBs typically activates NHEJ. However, a recent report suggests that a mutation of the Rev7 gene reduces NHEJ activity and stimulates HR [20]. Thus, intracellular NHEJ and HR activities tend to be either independent of one another or inversely correlated, and the inhibitory effect of olaparib is believed to reflect this association.

Furthermore, the current standard treatment for esophageal cancer is a combined platinum and radiation therapy with multifractionated radiotherapy involving a single dose of 1.8–2.0 Gy. Our study confirmed that even multidose irradiation of 2 Gy, which mimics actual esophageal cancer treatment, exhibits a high proton sensitization effect. Sensitizers in PBT have not been largely reported thus far, and the present study verifies for the first time a sensitization effect using 2 Gy multifractionated radiotherapy.

### Evaluation of combined olaparib and proton irradiation

To evaluate the biological effect of olaparib and proton irradiation, we assessed the dynamics of γ-H2AX foci, intensity and expression of DNA repair genes. DNA damage was observed to be increased in the proton-only and olaparib-only groups, and an additive increase in the olaparib and proton combination group was also observed (Fig. 6).

Notably, in the olaparib-PT group of OE-21 cells, there was no significant increase in the number of foci compared with the olaparib-only group, but there was a prominent increase in the intensity of the foci. Although the number of γ-H2AX foci is the most common indicator of DNA damage in radiation biology, the intensity of γ-H2AX foci is also frequently used [13, 16]. Because the significance of these two markers at a molecular level has not yet been established, their implications in this study cannot be concluded. However, we believe that there was a high possibility that DNA damage was increased in the olaparib-PT group compared with the olaparib-only group because the olaparib-PT group had lower colony-forming abilities and an increased expression of DNA repair genes.

In our study, we detected higher expression levels of three HR repair enzymes, i.e. Rad51, BRCA1 and BRCA2, in both the proton-only and olaparib-only groups (Fig. 7). Furthermore, the expression of these genes was increased additively using PBT in combination with olaparib. These findings indicate that both olaparib and PBT have biological effects on esophageal cancer cells via HR-dependent DSB repair. Recently, a sensitizing effect of olaparib on PBT has been reported in lung adenocarcinoma and pancreatic cancer cells. Thus, we concluded that the proton sensitization effect of olaparib occurs through increased DNA damage caused by G2/M cell cycle arrest and the conversion of sub-lethal non-DSB oxidative clustered DNA lesions (OCDLs) to lethal damage [11].

However, olaparib has been reported to exhibit a high sensitizing effect on photon therapy with an SER of 1.5–2.1 where there are no OCDLs [21]. Furthermore, if G2/M arrest is the primary effect, then both NHEJ- and HR-dependent DNA damage should accumulate; however, our results revealed the accumulation of only HR-dependent DNA damage (Fig. 7). From previous reports and our results, we hypothesize that the mechanism underlying the sensitizing effect of olaparib on PBT is an HR-dependent DSB accumulation rather than OCDL enhancement and cell arrest. Olaparib converts SSB into HR-dependent DNA damage through DNA amplification (Fig. 8A).

Radiation causes SSBs and DSBs (Fig. 8B). Although SSBs occur more frequently, they are easier to repair, and biological effects are primarily caused by DSBs [23]. Protons have been reported to cause more DSBs than photons. Our results indicate no difference between photon and proton sensitization. Therefore, we suggest that olaparib converts radiation-induced SSBs into HR-dependent DNA damage (Fig. 8C).

This mechanism suggests that PARP inhibitors may also be effective for cells that are resistant to platinum and photon therapy with high NHEJ activity. Recently, DNA damage drugs and radiation resistant is caused by high NHEJ activity and high copy number alterations can predict radiation resistance [17]. Furthermore, this study demonstrates that HR-dependent repair enzymes such as Rad51 and BRCA are important for repair following olaparib-PT, indicating the possibility that these enzymes could be used as biomarkers for olaparib-PT.

### Clinical trial of combined radiation therapy with olaparib

Olaparib offers a promising alternative because it helps reduce bone marrow suppression or mucosal damage, its dose-limiting toxicity does not overlap with radiation, and its anti-tumor effect is elevated even in hypoxic conditions that usually cause radiation resistance [7, 18, 24]. Notably, clinical trials involving a combination of olaparib and radiation therapy have already been initiated for breast cancer, lung cancer and head and neck cancer (NCT02229656, NCT01562210 and NCT02227082, respectively). Our results indicate that the combination of irradiation with olaparib can be clinically applied to esophageal cancer. Recent reports have suggested the efficacy of olaparib-RT in head and neck cancer, and its toxicity was also acceptable [25].

## CONCLUSION

We demonstrated that olaparib has a high sensitizing effect on PBT in platinum- and radiation-resistant esophageal cancer cells. The sensitizing effect was remarkable in multisplit irradiation, and an additive effect of HR-dependent DSB was considered the mechanism of action. Our findings suggest novel clinical applications of olaparib-PT against platinum and radiation-resistant esophageal cancer.

## CONFLICT OF INTEREST

The authors have no conflicts of interest.

## Supplementary Material

Fig_S1A_rrz088

Fig_S1B_rrz088

## References

[1] Lai EC, Mok FP, Tan ES et al. Endoscopic biliary drainage for severe acute cholangitis. N Engl J Med 1992;326:1582–6. 1584258 10.1056/NEJM199206113262401

[2] Cooper JS, Guo MD, Herskovic A et al. Chemoradiotherapy of locally advanced esophageal cancer: Long-term follow-up of a prospective randomized trial (RTOG 85-01). Radiation Therapy Oncology Group. JAMA 1999;281:1623–7. 10235156 10.1001/jama.281.17.1623

[3] Crosby T, Hurt CN, Falk S et al. Chemoradiotherapy with or without cetuximab in patients with oesophageal cancer (SCOPE1): A multicentre, phase 2/3 randomised trial. Lancet Oncol 2013;1:627–37. 10.1016/S1470-2045(13)70136-0 23623280

[4] Zhang P, Xi M, Li QQ et al. Concurrent cisplatin and 5-fluorouracil versus concurrent cisplatin and docetaxel with radiotherapy for esophageal squamous cell carcinoma: A propensity score-matched analysis. Oncotarget 2016;7:44686–94. 27183916 10.18632/oncotarget.9301 PMC5190128

[5] Minsky BD, Pajak TF, Ginsberg RJ et al. INT 0123 (radiation therapy oncology group 94-05) phase III trial of combined-modality therapy for esophageal cancer: High-dose versus standard-dose radiation therapy. J Clin Oncol 2002;20:1167–74. 11870157 10.1200/JCO.2002.20.5.1167

[6] Jiang Y, Verbiest T, Devery AM et al. Hypoxia potentiates the radiation-sensitizing effect of olaparib in human non-small cell lung cancer xenografts by contextual synthetic lethality. Int J Radiat Oncol Biol Phys 2016;95:772–81. 27020103 10.1016/j.ijrobp.2016.01.035 PMC4856738

[7] Mateo J, Carreira S, Sandhu S et al. DNA-repair defects and olaparib in metastatic prostate cancer. N Engl J Med 2015;373:1697–708. 26510020 10.1056/NEJMoa1506859 PMC5228595

[8] Gani C, Coackley C, Kumareswaran R et al. In vivo studies of the PARP inhibitor, AZD-2281, in combination with fractionated radiotherapy: An exploration of the therapeutic ratio. Radiother Oncol 2015;116:486–94. 26277432 10.1016/j.radonc.2015.08.003

[9] Paganetti H, Niemierko A, Ancukiewicz M et al. Relative biological effectiveness (RBE) values for proton beam therapy. Int J Radiat Oncol Biol Phys 2002;53:407–21. 12023146 10.1016/s0360-3016(02)02754-2

[10] Fontana AO, Augsburger MA, Grosse N et al. Differential DNA repair pathway choice in cancer cells after proton- and photon-irradiation. Radiother Oncol 2015;116:374–80. 26320609 10.1016/j.radonc.2015.08.014

[11] Hirai T, Saito S, Fujimori H et al. Radiosensitization by PARP inhibition to proton beam irradiation in cancer cells. Biochem Biophys Res Commun 2016;478:234–40. 27425251 10.1016/j.bbrc.2016.07.062

[12] Hojo H, Dohmae T, Hotta K et al. Difference in the relative biological effectiveness and DNA damage repair processes in response to proton beam therapy according to the positions of the spread out Bragg peak. Radiat Oncol 2017;12:111. 28673358 10.1186/s13014-017-0849-1 PMC5494883

[13] Schmid TE, Zlobinskaya O, Multhoff G Differences in phosphorylated histone H2AX foci formation and removal of cells exposed to low and high linear energy transfer radiation. Curr Genomics 2012;13:418–25. 23450137 10.2174/138920212802510501 PMC3426775

[14] Hosoya N, Miyagawa K Targeting DNA damage response in cancer therapy. Cancer Sci 2014;105:370–88. 24484288 10.1111/cas.12366 PMC4317796

[15] Jeggo PA, Geuting V, Lobrich M The role of homologous recombination in radiation-induced double-strand break repair. Radiother Oncol 2011;101:7–12. 21737170 10.1016/j.radonc.2011.06.019

[16] Ivashkevich A, Redon CE, Nakamura AJ et al. Use of the gamma-H2AX assay to monitor DNA damage and repair in translational cancer research. Cancer Lett 2012;327:123–33. 22198208 10.1016/j.canlet.2011.12.025 PMC3329565

[17] Yard BD, Adams DJ, Chie EK et al. A genetic basis for the variation in the vulnerability of cancer to DNA damage. Nat Commun 2016;7:11428. 27109210 10.1038/ncomms11428 PMC4848553

[18] Lin Y, McMahon SJ, Paganetti H et al. Biological modeling of gold nanoparticle enhanced radiotherapy for proton therapy. Phys Med Biol. 2015;60:4149–68. 25953956 10.1088/0031-9155/60/10/4149

[19] Hojo H, Dohmae T, Hotta K et al. Effect of 5-fluorouracil on cellular response to proton beam in esophageal cancer cell lines according to the position of spread-out Bragg peak. Acta Oncol. 2019;58:475–82. 30632869 10.1080/0284186X.2018.1555373

[20] Xu G, Chapman JR, Brandsma I et al. REV7 counteracts DNA double-strand break resection and affects PARP inhibition. Nature 2015;521:541–4. 25799992 10.1038/nature14328 PMC4671316

[21] Jang NY, Kim DH, Cho BJ et al. Radiosensitization with combined use of olaparib and PI-103 in triple-negative breast cancer. BMC Cancer 2015;15:89. 25884663 10.1186/s12885-015-1090-7 PMC4355140

[22] Verhagen CV, de Haan R, Hageman F et al. Extent of radiosensitization by the PARP inhibitor olaparib depends on its dose, the radiation dose and the integrity of the homologous recombination pathway of tumor cells. Radiother Oncol 2015;116:358–65. 25981132 10.1016/j.radonc.2015.03.028

[23] Girdhani S1, Sachs R, Hlatky L Biological effects of proton radiation: What we know and don't know. Radiat Res 2013;179:257–72.27. 23373900 10.1667/RR2839.1

[24] Ledermann J, Harter P, Gourley C et al. Olaparib maintenance therapy in platinum-sensitive relapsed ovarian cancer. N Engl J Med 2012;366:1382–92. 22452356 10.1056/NEJMoa1105535

[25] Karam SD, Reddy K, Blatchford PJ et al. Final report of a phase I trial of Olaparib with Cetuximab and radiation for heavy smoker patients with locally advanced head and neck cancer. Clin Cancer Res 2018;24:4949–59. 30084837 10.1158/1078-0432.CCR-18-0467 PMC6873707

