# Supplementary figures and images for: PARP inhibitor olaparib sensitizes esophageal carcinoma cells to fractionated proton irradiation
Source: J Radiat Res. 2020 Jan 24;61(2):177–86. doi: 10.1093/jrr/rrz088 (PMC7246074; doi:10.1093/jrr/rrz088)

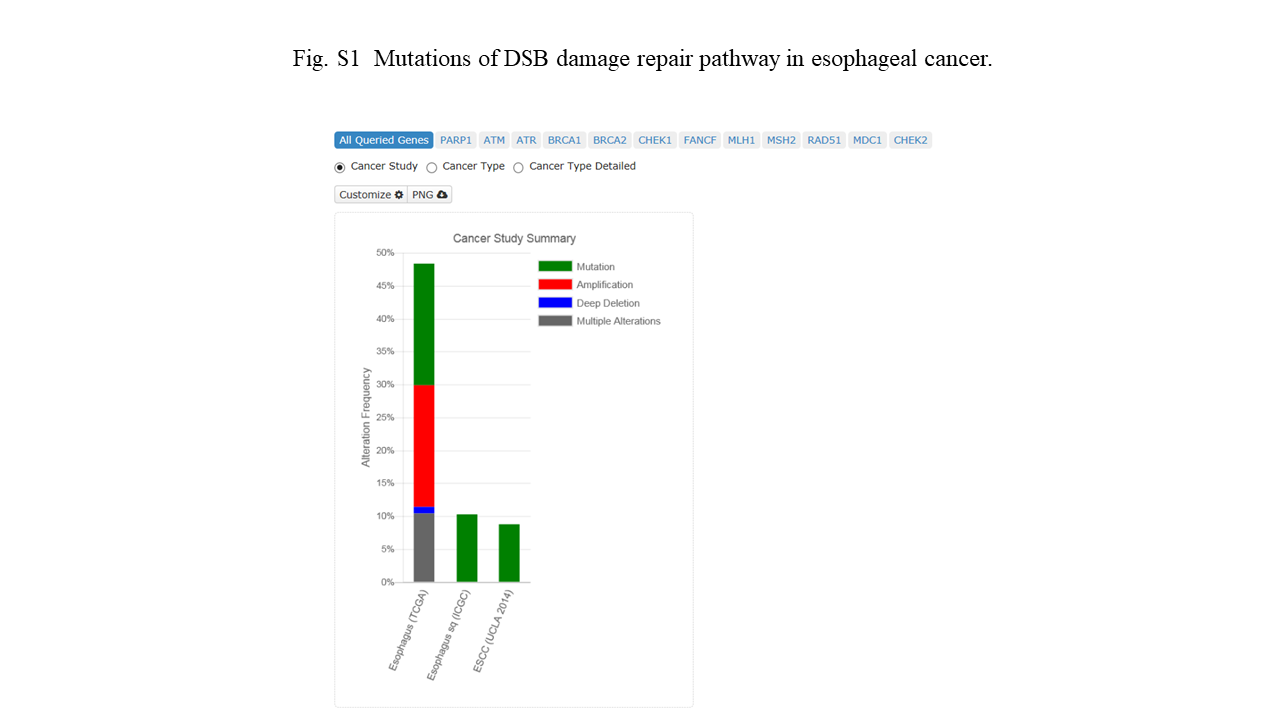

Supplement: Fig_S1A_rrz088 [file fig_s1a_rrz088.png]

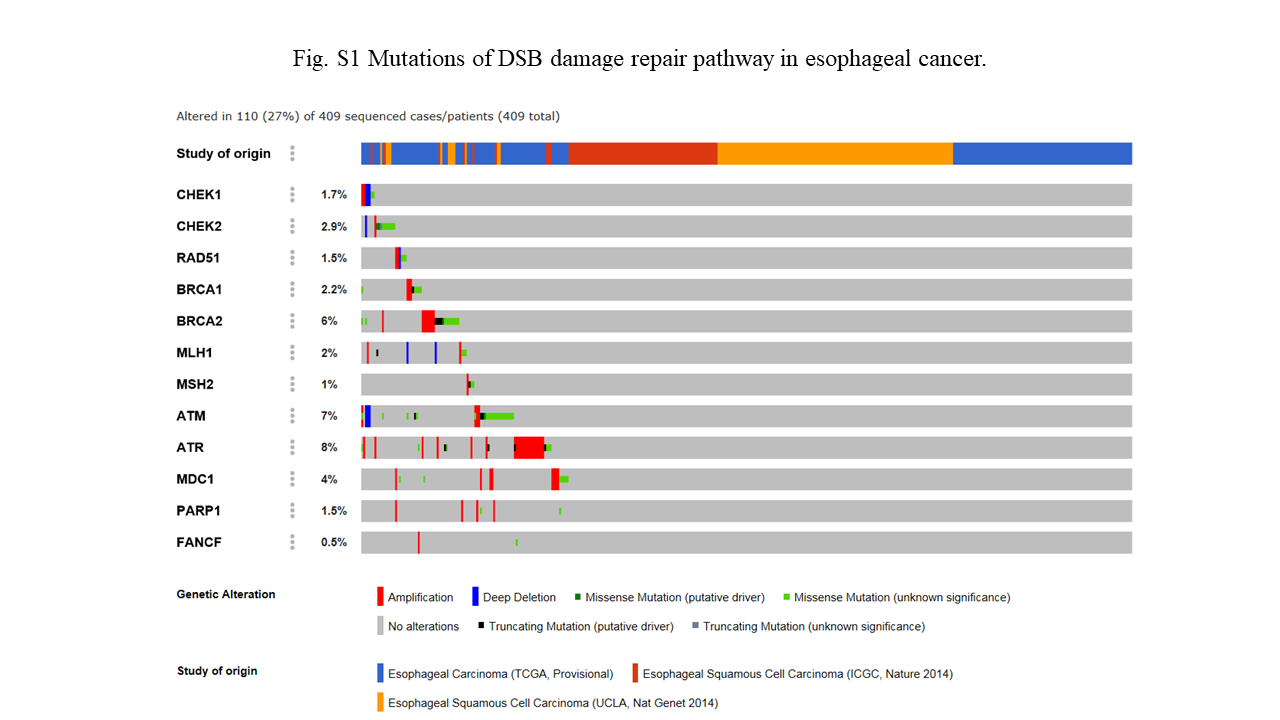

Supplement: Fig_S1B_rrz088 [file fig_s1b_rrz088.png]
